# Functional interleukin‐6 receptor‐α is located in tanycytes at the base of the third ventricle

**DOI:** 10.1111/jne.12546

**Published:** 2017-12-12

**Authors:** F. Anesten, C. Santos, E. Gidestrand, E. Schéle, V. Pálsdóttir, T. Swedung‐Wettervik, B. Meister, K. Patrycja Skibicka, J.‐O. Jansson

**Affiliations:** ^1^ Department of Physiology Institute of Neuroscience and Physiology the Sahlgrenska Academy at the University of Gothenburg Gothenburg Sweden; ^2^ Department of Neuroscience Karolinska Institutet Stockholm Sweden; ^3^ Wallenberg Centre for Molecular and Translational Medicine University of Gothenburg Sweden

**Keywords:** hypothalamus, IL‐6, IL‐6Rα, tanycytes, third ventricle

## Abstract

Interleukin (IL)‐6^−^/^−^ mice develop mature onset obesity, whereas i.c.v. injection of IL‐6 decreases obesity in rodents. Moreover, levels of IL‐6 in cerebrospinal fluid (CSF) were reported to be inversely correlated with obesity in humans. Tanycytes lining the base of the third ventricle (3V) in the hypothalamus have recently been reported to be of importance for metabolism. In the present study, we investigated whether tanycytes could respond to IL‐6 in the CSF. With immunohistochemistry using a well characterised antibody directed against the ligand binding receptor for IL‐6, IL‐6 receptor α (IL‐6Rα), it was found that tanycytes, identified by the two markers, vimentin and dopamine‐ and cAMP‐regulated phosphoprotein of 32 kDa, contained IL‐6Rα. There were fewer IL‐6Rα on another type of ventricle‐lining cells, ependymal cells, as identified by the marker glucose transporter‐1. To demonstrate that the immunoreactive IL‐6Rα were responsive to IL‐6, we injected IL‐6 i.c.v. This treatment increased immunoreactive phosphorylated signal transducer and activator of transcription‐3 (pSTAT3) in tanycytes after 5 minutes and in cells in the medial part of the arcuate nucleus after 5 and 15 minutes. Intracerebroventricular injection of leptin exerted similar effects. As expected, i.p. injection of leptin also induced pSTAT3 staining in the hypothalamus, whereas i.p. IL‐6 injection had little effect on this parameter. Intracerebroventricular or i.p. injection of vehicle only had no effect on pSTAT3‐immunoreactivity. In summary, there are functional IL‐6Rα on tanycytes at the bottom of the 3V, in agreement with the possibility that ventricular administration of IL‐6 decreases obesity in mice via an effect on this cell type.

## 1 INTRODUCTION

Interleukin‐6 (IL‐6) has been studied extensively in the field of immunology, and is essentially regarded as a classic pro‐inflammatory cytokine, together with, for example, IL‐1 and tumour necrosis factor‐α, although IL‐6 may also occasionally have anti‐inflammatory properties.^1^, ^2^ During inflammation and catabolic conditions, IL‐6 is often found in the circulation at supraphysiological concentrations, and can then suppress food intake, stimulate energy expenditure and even enhance core body temperature.^3^, ^4^, ^5^ During health, IL‐6 in lower more physiological, doses appears to consistently decrease body fat mass and increase metabolism.^3^, ^6^, ^7^, ^8^, ^9^ Several research groups, including ours, have reported that IL‐6^−^/^−^ mice develop increased fat mass from approximately 6 months of age.^7^, ^8^, ^10^ This fat mobilising action of IL‐6 in mice and rats may be exerted at the level of the central nervous system (CNS), probably the hypothalamus and/or the brainstem.^6^, ^10^ A lack of IL‐6 could dampen the anti‐obesity effect of leptin, whereas brain‐specific overproduction of IL‐6 may enhance the effect of leptin.^11^, ^12^

The results of earlier studies indicate that the obesity reducing action of IL‐6 is primarily exerted in the brain.^6^, ^10^, ^12^, ^13^ Therefore, it could be of value to identify cells that contain interleukin‐6 receptor‐α (IL‐6Rα) and determine whether these cells are located in regions that influence food intake and energy balance. Earlier studies have indicated that IL‐6Rα is present on neurons in arcuate nucleus (ARC), paraventricular nucleus (PVN) and lateral hypothalamic area (LHA) of the hypothalamus, as well as the nucleus of the solitary tract (NTS) in the hindbrain.^14^, ^15^, ^16^, ^17^

Intracerebroventricular administration of IL‐6 to experimental animals decreases body fat and increases energy expenditure and thermogenesis,^6^, ^10^, ^18^, ^19^ whereas systemic administration of IL‐6 is less effective.^6^, ^10^ Moreover, IL‐6 levels in the cerebrospinal fluid (CSF) of humans has been reported to be inversely correlated with body fat mass.^20^ Taken together, these studies are in agreement with the assumption that exogenous and endogenous IL‐6 in the ventricle system can play a role in metabolic regulation. If this is the case, one obvious and logical mechanism for how ventricle IL‐6 could influence energy‐regulating nuclei could be to target ventricle‐lining cells.

Tanycytes are hypothalamic radial glia‐like cells lining the ventricle walls, especially at the bottom of the third ventricle (3V).^21^ This cell type has recently been implicated in the regulation of feeding and energy balance.^22^, ^23^, ^24^

In the present study, we investigated whether IL‐6Rα is located in tanycytes. To demonstrate that immunoreactive IL‐6Rα were responsive to IL‐6, we injected IL‐6 i.c.v. or i.p. and then investigated whether IL‐6 induced phosphorylated signal transducer and activator of transcription‐3 (pSTAT3) immunoreactivity in tanycytes. We also measured pSTAT3‐immunoreactivity in the ARC, an area previously found to contain IL‐6Rα.^16^ For comparison we measured immunoreactive pSTAT3 after i.c.v. or i.p. administration of leptin, a well‐known inducer of pSTAT3 in tanycytes and ARC.^23^

## 2 MATERIALS AND METHODS

### 2.1 Animals

C57BL6 male and female wild‐type mice, aged 4‐6 months of age (Jackson Laboratories, Bar Harbor, ME, USA), were used for the immunohistochemical staining. Animals had free access to water and standard chow pellets (Tekland Global, Harlan, the Netherlands) and were kept under a 12:12 hours light/dark cycle (lights on 06.00 hours), 50%‐60% relative humidity at 24‐26°C temperature. All animal procedures were approved by the local ethics committee for animal care at the University of Gothenburg, and were conducted in accordance with their guidelines.

### 2.2 Tissue preparation for immunohistochemistry

Mice were anaesthetised with a rompun‐ketamine mixture and perfused transcardially with heparinised saline (50 IU mL^‐1^) followed by 4% paraformaldehyde in 0.1 mol L^‐1^ phosphate buffer. The brains were removed and post‐fixed in 4% paraformaldehyde in 0.1 mol L^‐1^ phosphate buffer containing 15% sucrose overnight at 4°C. They were then transferred to a 30% sucrose solution in 0.1 mol L^‐1^ phosphate buffer until sectioning.

Coronal sections (thickness 20 μm) of the hypothalamus (Bregma −1.70 to −1.94, interneural 2.10 to 1.86) were cut using a CM3050S cryostat (Leica Microsystem, Wetzlar, Germany) and stored in tissue storage solution (25% ethylene glycol, 25% glycerol, 50% 0.1 mol L^‐1^ phosphate buffer).

### 2.3 Immunohistochemistry

Sections were rinsed in Tris‐NaCl‐Triton‐X (TNT) washing buffer (0.1 mol L^‐1^ TrisHCl, pH 7.5, 0.15 mol L^‐1^ NaCl, 0.2% Triton‐X‐100) and then blocked for 1 hour with Tris‐NaCl‐blocking buffer (TNB) (Perkin Elmer, Waltham, MA, USA). Sections were then incubated with primary antibodies against IL‐6Rα along with either antibodies against dopamine‐ and cAMP‐regulated phosphoprotein of 32 kDa (DARPP‐32), vimentin, glial fibrillary acidic protein (GFAP), pSTAT3 or glucose transporter‐1 (GLUT‐1) (Table 1) for 2 days in 4°C. After rinsing, sections were incubated for 1 hour with secondary antibodies (Table 1) diluted in TNB blocking solution (0.1 mol L^‐1^ Tris‐HCl (pH 7.5), 0.15 mol L^‐1^ NaCl, 0.5% blocking reagent (FP1020; Perkin Elmer). Sections were rinsed in TNT washing buffer and the IL‐6Rα signal was developed by incubating the sections in rabbit anti‐rat horseradish peroxidase in TNB blocking reagent (dilution 1:100, TSA System; Perkin Elmer) for 1 hour. After amplification, they were stained with fluorescein tyramide (dilution 1:50, TSA System; Perkin Elmer) in amplification diluent (TSA System; Perkin Elmer) for 10 minutes. Sections were then rinsed in washing buffer and the nuclei were stained with 4’,6‐diamidino‐2‐phenylindole (DAPI) (dilution 1:5000, D1306; Molecular Probes, Carlsbad, CA, USA) for 5 minute, rinsed in TNT washing buffer without 0.2% Triton‐X‐100 and mounted on slides (SuperFrost^®^Plus, 4951PLUS; Thermo Scientific, Waltham, MA, USA) with prolong gold anti‐fade (P36930; Molecular Probes).

**Table 1:** Basic information about the primary and secondary antibodies used in the present study

Antiserum	Dilution	Catalogue number	Manufacturer
Rat anti‐IL‐6Rα	1:20	MAB18301	R&D Systems, Minneapolis, MN, USA
Rabbit anti‐GFAP	1:200	Z 0334	DakoCytomation, Glostrup, Denmark
Mouse anti‐DARPP‐32	1:100	2308	Kind gift from Professor Paul Greengard, Rockefeller University, New York, NY, USA^50^
Goat anti‐GLUT‐1	1:200	sc‐1605	Santa Cruz Biotechnology, Santa Cruz, CA, USA
Chicken anti‐Vimentin	1:200	AB5733	Merck Millipore, Billerica, MA, USA
Rabbit anti‐Phospho‐Stat3	1:100	#9145S	Cell Signaling Technology, Danvers, MA, USA
Rabbit anti‐IL‐6Rα	1:200	SC‐660	Santa Cruz Biotechnology, Santa Cruz, CA, USA
Goat anti‐mouse Alexa fluor 568	1:250	A‐11031	Molecular Probes, Carlsbad, CA, USA
Goat anti‐chicken Alexa fluor 594	1:250	A‐11042	Molecular Probes, Carlsbad, CA, USA
Goat anti‐rabbit Alexa fluor 488	1:250	A‐11008	Molecular Probes, Carlsbad, CA, USA
Goat anti‐rabbit Alexa fluor 568	1:250	A‐11036	Molecular Probes, Carlsbad, CA, USA

Sections were incubated with secondary antibodies only, or with mismatching primary and secondary antibodies, resulting in negative staining as a control for unwanted cross‐reactivity. To confirm the identification of tanycytes, two different antibodies were used; DARPP‐32^25^, ^26^ and vimentin (Table 1). As a control for the IL‐6Rα‐antibody against the extracellular domain used in the main study, a second IL‐6Rα‐antibody against the intracellular domain was used as a control (see Supporting information, Figure S1).

### 2.4 Confocal microscopy and cell counting

Images of the stained sections were obtained using either a confocal microscope system (LSM 700; Carl Zeiss, Oberkochen, Germany), together with a Plan APO ×63 A/1.40 oil lens (for close‐up images) or a Plan Fluor ×20/0.75 lens (for anatomical overview pictures) and a solid‐state laser. Images for each stain were taken in series with the same exposure times and light settings for each slide. The pinhole was set to 1 airy unit. Images were then processed using fiji software (http://fiji.sc/Fiji) using the same settings for contrast and brightness for all slides. Brain sections were then averaged for each animal and representative images chosen for cell counting.

The IL6‐Rα‐, vimentin‐, GLUT‐1‐, pSTAT3‐ and/or DARPP32‐labelled cells were visualised with a confocal microscope (LSM 700; Carl Zeiss). For all instances of cell counting, results were calculated from two brains, two sections per brain and averaged per animal. Triple channel confocal images (to cover the entire section of the location of interest) were generated with a Plan Fluor ×20/0.75 lens and a solid‐state laser.

A tile scan of 300×300 μm was obtained from the medial ARC corresponding to a representative part of the nucleus. For 3V wall counting, only vimentin‐immunoreactive cells that were within the anatomical distribution of β‐tanycytes in the plane of image were counted. For ependymal cell counting, only cells dorsal of β‐tanycytes in the plane of image were counted. For subependymal cell counting, only GFAP‐positive cells in the subependymal layer below β‐tanycytes in the plane of image were counted. Cells were considered IL6‐Rα‐, vimentin‐, GLUT‐1‐, pSTAT3‐ and/or DARPP32‐labelled when the staining was clearly above background and their DAPI‐labelled cell nuclei were in the plane of image. Co‐localisation was determined by switching between red‐, green‐ and blue‐channel images.

### 2.5 Intracerebroventricular and i.p. administration of IL‐6 or leptin

Mice were anaesthetised with isoflurane (Baxter, Deerfield, IL, USA) and fixed in a model 900 stereotax (Kopf [Bilaney Consultants]; St Julians, UK). Depth for Bregma and lambda was found and the skull adjusted until both areas were at the same depth. Coordinates for Bregma were found and the injection pump (KDS‐310‐PLUS; GENEQ Inc., Anjou, QC, Canada) holding the injection syringe (Hamilton, Reno, NV, USA) was moved −1.0 mm laterally and −0.3 mm posteriorly of Bregma. A hole was drilled to allow passage of the injection needle into the lateral ventricle. The injection needle was inserted −2.4 mm below the depth of bregma and 1.5 μL of recombinant rat IL‐6 (40 ng per mouse; PeproTech, Rocky Hill, NJ, USA) or recombinant murine leptin (1 mg kg^‐1^; PeproTech) in sterile artificial cerebrospinal fluid (aCSF) or aCSF was injected over 2 minutes. The needle was allowed to stay in the ventricle for 2 more minutes before being slowly withdrawn. Mice were anaesthetised with domitor‐ketamine and perfused as above 5 or 15 minutes after injection. aCSF administration was used as a negative control and resulted in an absence of pSTAT3‐immunoreactivity (not shown).

For i.p. injection, mice were injected with 100 μL of recombinant rat IL‐6 (80 ng per mouse; PeproTech) or recombinant murine leptin (3 mg kg^‐1^; PeproTech) in sterile NaCl or NaCl only. To confirm that i.p. administration of IL‐6 was successful, we measured pSTAT3‐immunoreactivity in the subfornical organ, a part of the brain outside the blood‐brain barrier, as a positive control (see Supporting information, Figure S3). NaCl administration was used as a negative control and resulted in the absence of pSTAT3‐immunoreactivity (not shown).

The dose of IL‐6 given i.p. was chosen from previous studies and is intended to be physiological rather than pathophysiological. The dose of IL‐6 with respect to body weight given i.p. as reported by Wallenius et al.,^10^ and resulted in serum IL‐6 levels much lower than those observed after after lipopolysaccharide treatment.

Mice were anaesthetised with domitor‐ketamine and perfused as above 15 minutes after injection. Injection doses of IL‐6 i.c.v.,^6^ leptin i.c.v.,^27^ leptin i.p.^23^ and IL‐6 i.p.^10^ were selected in accordance with previous studies.

## 3 RESULTS

### 3.1 IL‐6Rα‐immunoreactivity is located in vimentin‐ or DARPP‐32‐immunoreactive cells lining the floor and ventral walls of the 3V

**Figure 1:**
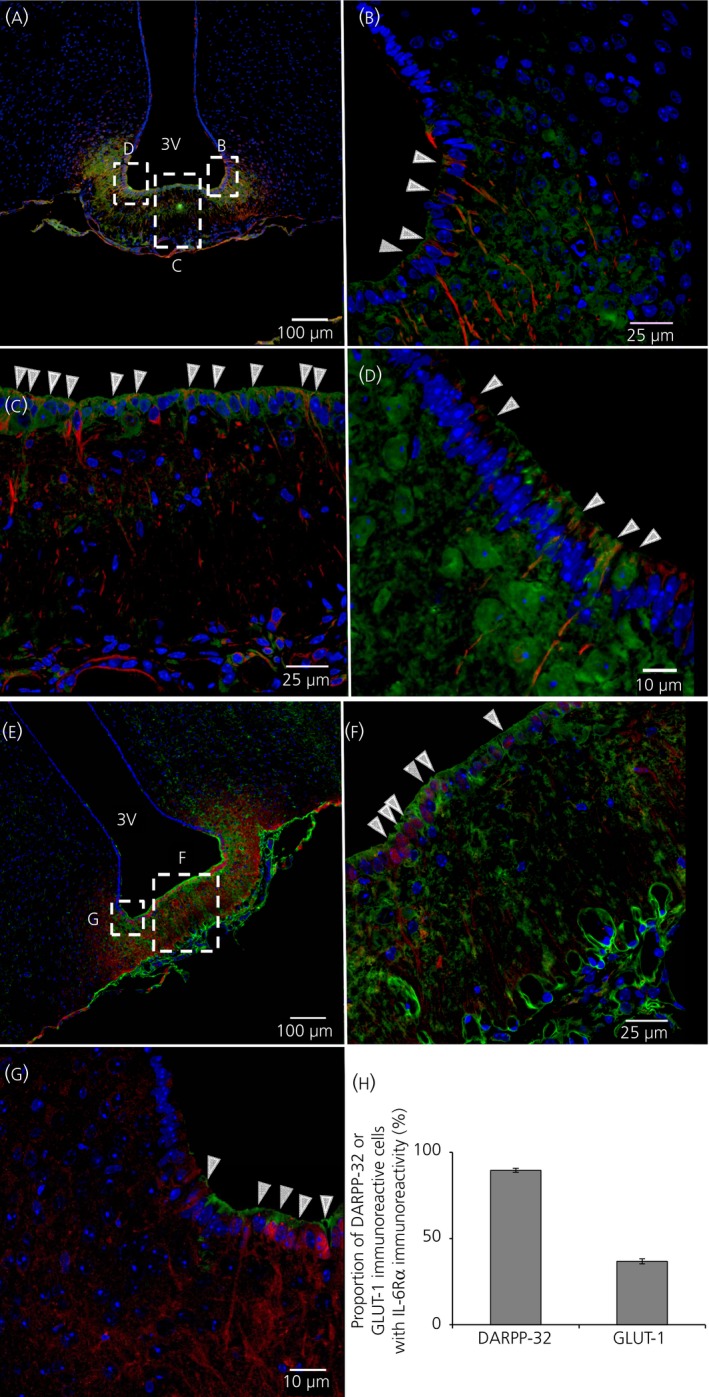
Interleukin (IL)‐6Rα immunoreactivity is located in tanycytes bordering the median eminence. Immunohistochemistry showing IL‐6 receptor α‐ (IL‐6Rα) immunoreactivity in green, vimentin‐ (A‐D) or dopamine‐ and cAMP‐regulated phosphoprotein of 32 kDa (DARPP‐32) (E‐G) immunoreactivity in red and 4’,6‐diamidino‐2‐phenylindole (DAPI) (nuclear staining) in blue, shown in coronal cross‐sections of parts of the third ventricle (3V) of the mouse brain. An overview of the area (A, E) and magnifications of parts of the 3V facing the median eminence (ME) (C, F) and arcuate nucleus (ARC) (B, D, G) showed IL‐6Rα‐immunoreactivity on vimentin‐ or DARPP‐32‐immunoreactive cells. White arrowheads (B‐D, F‐G) indicate examples of such cells displaying both vimentin‐ or DARPP‐32‐ and IL‐6Rα‐immunoreactivity. Approximately 90% of DARPP‐32‐immunoreactive cells also showed IL‐6Rα‐immunoreactivity, whereas only approximately 35% of GLUT1‐immunoreactive cells showed IL‐6Rα‐immunoreactivity (H). Images were obtained using the confocal microscope system described in the Materials and methods. Cell counting was performed from two animals using two slices per animal as described in the Materials and methods. Scale bars (overview)=100 μm, close‐up=25 μm (B, C, F, G) or 10 μm (D)

Figure 1A‐D shows that vimentin immunoreactive cells (in red) have a characteristic distribution along the 3V wall. These cells aggregate towards the basal parts of the ventricle wall with a few spread cells along the lateral walls (Figure 1A). This is in good agreement with the distribution reported for β‐tanycytes^21^ and vimentin has previously been used as a marker for tanycytes.^28^, ^29^ A significant proportion of these vimentin‐immunoreactive cells lining the basal ventricle wall show IL‐6Rα‐immunoreactivity (in green) (Figure 1B‐D, examples indicated by white arrowheads). To strengthen the data shown in Figure 1, DARPP‐32, another marker for tanycytes, was co‐stained with IL‐6Rα (Figure 1E‐G). This antibody displayed a similar pattern of immunoreactivity as the antibody against vimentin, with many cells showing both DARPP‐32‐ and IL‐6Rα‐immunoreactivity (Figure 1F,G, white arrowheads). Cell counting showed that approximately 90% of the DARPP‐32‐immunoreactive cells were also IL‐6Rα‐immunoreactive (Figure 1H). In agreement with this, approximately 90% of the vimentin‐immunoreactive cells lining the basal ventricle walls also displayed IL‐6Rα‐immunoreactivity (not shown). Taken together, our data indicated that a large proportion of vimentin‐ or DARPP‐32‐immunoreactive cells at the bottom of 3V, presumably representing β‐tanycytes, are also IL‐6Rα‐immunoreactive.

### 3.2 IL‐6Rα‐immunoreactivity is present, to a lesser degree, on GLUT‐1‐immunoreactive ependymal cells at the lateral wall of the 3V

**Figure 2:**
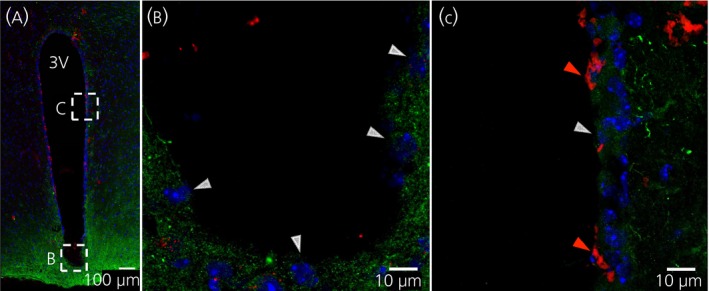
Interleukin (IL)‐6Rα immunoreactivity is located in some glucose transporter‐1 (GLUT‐1)‐positive ependymal cells. Immunohistochemistry showing IL‐6 receptor α‐ (IL‐6rα) immunoreactivity in green, GLUT‐1 (a marker for ependymal cells) immunoreactivity in red and 4’,6‐diamidino‐2‐phenylindole (DAPI) (nuclear staining) in blue, shown in coronal cross‐sections of parts of the third ventricle (3V) of the mouse brain. Overview of the area (A) and magnifications of parts of the 3V facing the arcuate nucleus (ARC) (B) and lateral 3V wall (C) showed IL‐6Rα immunoreactivity on some of the GLUT‐1 immunoreactive cells. Red arrowheads (C) indicate examples of such cells with both GLUT‐1‐ and IL‐6Rα‐immunoreactivity, whereas white arrowheads indicate cells with only IL‐6Rα‐immunoreactivity (B, C). Images were obtained using the confocal microscope system described in the Materials and methods. Scale bars (overview)=100 μm, close‐up=10 μm

Co‐staining of IL‐6Rα with GLUT‐1 (used here as a marker of ependymal cells of the blood‐brain barrier) (Figure 2) showed a different pattern from the vimentin‐ or DARPP‐32‐immunoreactivity (Figure 1). Some of the GLUT‐1‐immunoreactive cells (red) in the lateral wall of the 3V also showed IL‐6Rα immunoreactivity (green) (Figure 2C, red arrowheads). By contrast, cells at the ventral part of the 3V lateral wall showed only IL‐6Rα‐immunoreactivity without GLUT‐1‐immunoractivity (Figure 2C, white arrowheads). Indeed, cell counting showed that approximately 35% of the GLUT‐1‐immunoreactive cells also showed IL‐6Rα‐immunoreactivity (Figure 1H). Taken together, these results indicate that the IL‐6Rα is mainly located in vimentin‐ or DARPP‐32‐immunoreactive 3V lining cells, presumably tanycytes, bordering the median eminence and ARC. This differs from the sparse IL‐6Rα‐immunoreactivity observed in GLUT‐1‐immunoreactive cells.

### 3.3 IL‐6 or leptin induced pSTAT3‐immunoreactivity 5 minute after i.c.v. injection in ventricle lining vimentin‐immunoreactive cells

**Figure 3:**
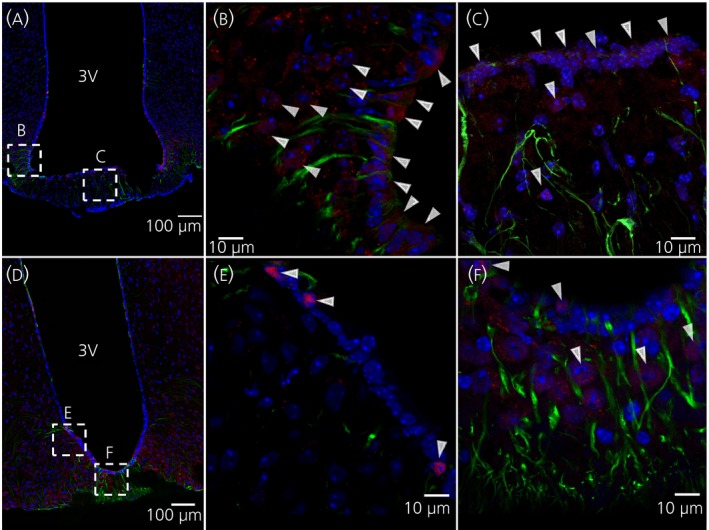
Intracerebroventricular administration of leptin or interleukin (IL)‐6 induces pSTAT3 immunoreactivity in tanycytes after 5 minutes. Immunohistochemistry showing phosphorylated signal transducer and activator of transcription‐3 (pSTAT3)‐immunoreactivity in red, vimentin‐ (a tanycyte marker) immunoreactivity in green and 4’,6‐diamidino‐2‐phenylindole (DAPI) (nuclear staining) in blue, shown in coronal cross‐sections of parts of the third ventricle (3V) of the mouse brain. Images were obtained from mice that were killed 5 minute after i.c.v. injection of IL‐6 (A‐C) or leptin (D‐F). White arrowheads indicate examples of pSTAT3‐immunoreactivity. Images were obtained using the confocal microscope system described in the Materials and methods. Scale bars for overview=100 μm, close‐up=10 μm

Figure 3 shows immunohistochemistry of the ventral 3V, 5 minute after i.c.v. injection of IL‐6 (Figure 3A‐C) or leptin (Figure 3D‐f). pSTAT3 is used as a marker for intracellular signalling. White arrowheads indicate pSTAT3‐immunoreactivity in ventricle‐lining vimentin‐immunoreactive cells (Figure 3B,E) and the median eminence (ME) (Figure 3C,F).

**Figure 4:**
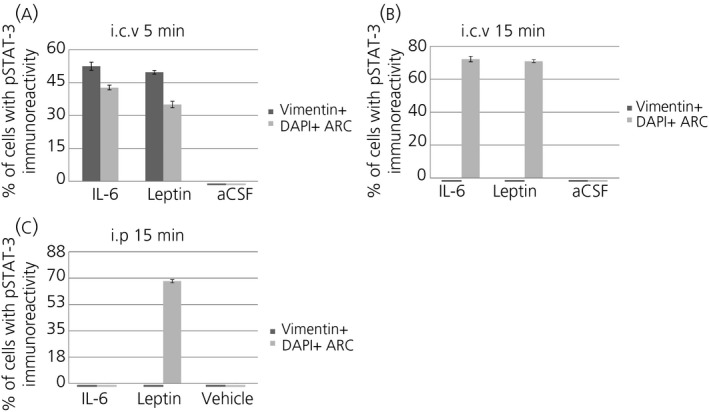
Quantification of phosphorylated signal transducer and activator of transcription‐3 (pSTAT3)‐immunoreactivity after i.c.v. and i.p. administration of interleukin (IL)‐6, leptin or vehicle. Mice killed 5 minutes after i.c.v. administration of IL‐6 or leptin (A) show pSTAT3‐immunoreactivity in approximately 52% or 49% of ventricle‐lining vimentin‐positive cells, respectively. This immunoreactivity is absent in mice killed 15 minutes after i.c.v. administration of these substances (B). In the arcuate nucleus (ARC), i.c.v. IL‐6 administration induces pSTAT3‐immunoreactivity in approximately 43% of cells after 5 minutes (A) and 72% (B) after 15 minutes, whereas leptin similarly induces pSTAT3‐immunoreactivity in approximately 35% of cells after 5 minutes (A) and 71% of cells after 15 minutes (B). Intraperitoneal administration of IL‐6 does not induce pSTAT3‐immunoreactivity in the ARC after 15 minutes, whereas leptin induces pSTAT3‐immunoreactivity in approximately 68% of ARC cells (C). Articifial cerebrospinal fluid (aCSF) (A‐B) or saline (C) does not induce pSTAT3‐immunoreactivity in either ventricle‐lining vimentin‐immunoreactive cells or ARC cells. Cell counting was performed from two animals using two slices per animal as described in the Materials and methods

Cell counting shows that approximately 52% and 49% of the vimentin‐immunoreactive cells also showed pSTAT3‐immunoreactivity in mice treated with i.c.v. IL‐6 and leptin, respectively (Figure 4A). Approximately 43% or 35% of the cells in the ARC were positive for pSTAT3 in mice treated with i.c.v. IL‐6 and leptin, respectively (Figure 4A).

### 3.4 Intracerebroventicular administered IL‐6 or leptin induced pSTAT3‐immunoreactivity in ARC cells 15 minute after injection

**Figure 5:**
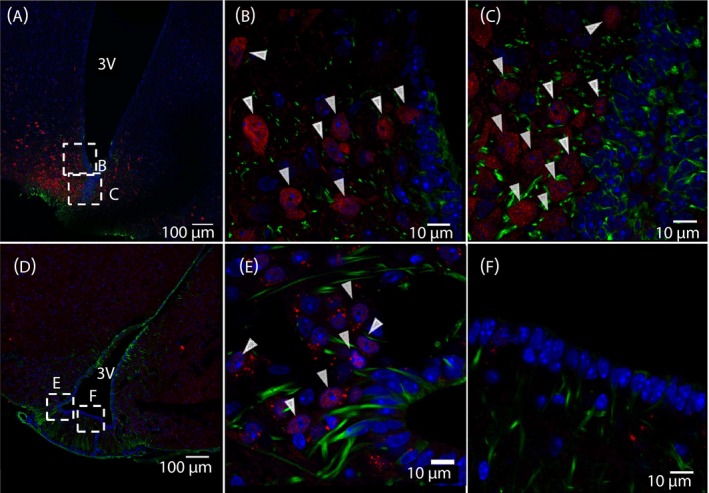
Intracerebroventricular administration of leptin or interleukin (IL)‐6 induces phosphorylated signal transducer and activator of transcription‐3 (pSTAT3)‐immunoreactivity in the arcuate nucleus (ARC) after 15 minutes. Immunohistochemistry showing pSTAT3‐immunoreactivity in red, vimentin‐ (a tanycyte marker) immunoreactivity in green and 4’,6‐diamidino‐2‐phenylindole (DAPI) (nuclear staining) in blue, shown in coronal cross‐sections of parts of the third ventricle (3V) of the mouse brain. Images were obtained from mice that were killed 15 minutes after i.c.v. injection of IL‐6 (A‐C) or leptin (D‐F). White arrowheads indicate examples of pSTAT3‐immunoreactivity in the ARC. Images were obtained using the confocal microscope system described in the Materials and methods. Scale bars (overview)=100 μm, close‐up=10 μm

Figure 5 shows immunohistochemistry of the ventral 3V, 15 minutes after i.c.v. injection of IL‐6 (Figure 5 a‐c) or leptin (Figure 5 d‐f). White arrowheads indicate pSTAT3 immunoreactivity in the ARC (Figure 5 b,c,e). Cell counting shows that approximately 72% and 71% of ARC cells were also positive for pSTAT3 in mice treated 15 minutes earlier with i.c.v. IL‐6 and leptin, respectively (Figure 4B). By contrast, no ventricle‐lining vimentin‐positive cells showed pSTAT3‐immunoreactivity 15 minutes after i.c.v. injection of IL‐6 or leptin (Figures 4B and 5C,F).

### 3.5 Intraperitoneal administration of leptin, but not IL‐6, increased pSTAT3‐immunoreactivity in the ARC after 15 minute

**Figure 6:**
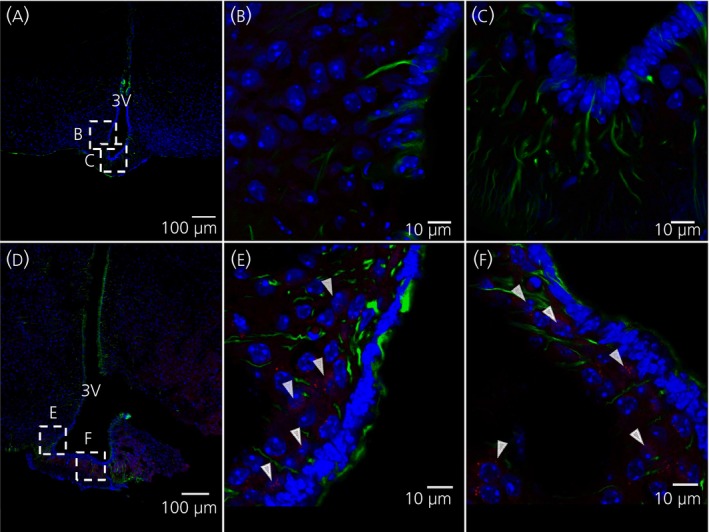
Systemic leptin, but not interleukin (IL)‐6, induces phosphorylated signal transducer and activator of transcription‐3 (pSTAT3) activation in the arcuate nucleus (ARC) after 15 minutes. Immunohistochemistry showing pSTAT3‐immunoreactivity in red, vimentin‐ (a tanycyte marker) immunoreactivity in green and 4’,6‐diamidino‐2‐phenylindole (DAPI) (nuclear staining) in blue, shown in coronal cross‐sections of parts of the third ventricle (3V) of the mouse brain. Images were obtained from mice that were killed 15 minutes after i.c.v. injection of IL‐6 (A‐C) or leptin (D‐F). White arrowheads indicate examples of pSTAT3‐immunoreactive cells. Images were obtained using the confocal microscope system described in the Materials and methods. Scale bars (overview)=100 μm, close‐up=10 μm2

Figure 6 shows immunohistochemistry of the ventral 3V wall, 15 minute after i.p. injection of IL‐6 (Figure 6 a‐c) or leptin (Figure 6 d‐f). There is a high proportion of ARC cells with DAPI‐immunoreactive cell nuclei that show pSTAT3 immunoreactivity (in red, white arrowheads) after i.p. administration of leptin (Figure 6 d‐f). There was no such effect in the group treated with IL‐6 (Figure 6 a‐c). Cell counting shows that approximately 69% of DAPI‐immunoreactive ARC cells were also positive for pSTAT3 in mice treated i.p. 15 minute earlier with leptin. By contrast, no cells were positive for pSTAT3 in mice treated i.p. with IL‐6 (Figure 4C) or vehicle [not shown]).

## 4 DISCUSSION

The results of the present study indicate that functional ligand binding IL‐6Rα are located in ventricle lining cells of the 3V in mice, especially on β‐tanycytes at the bottom of the 3V. This group of tanycytes lies close to the ME and ARC and has recently been shown to be of importance for energy balance and metabolism.^23^ Tanycytes in the lateral 3V wall are interspersed with ependymal cells that show GLUT‐1‐immunoreactivity.^30^ In the present study, IL‐6Rα‐immunoreactivity in GLUT1 positive ependymal cells was scarce in comparison with tanycytes. Intracerebroventricular injection of IL‐6 increased STAT3 phosphorylation, a well‐known intracellular mediator step in IL‐6 signalling,^31^ in tanycytes after 5 minutes. The latter finding indicates that the IL‐6Rα on tanycytes are functional.

There is a growing body of evidence that IL‐6 can decrease body fat mass via effects in the brain. Previous studies have shown that deficiency of IL‐6 is causing mature onset obesity,^7^, ^8^, ^10^ and the results of several studies involving central IL‐6 administration indicate that this anti‐obesity effect is exerted at the level of the brain.^6^, ^20^, ^32^ In addition, there is evidence that brain IL‐6 is crucial in the anti‐obesity signalling of both GLP‐1 analogues and amylin analogues.^33^, ^34^, ^35^, ^36^ These functions of IL‐6 are of clinical relevance, given that both GLP‐1‐ and amylin analogues are widely used for treatment of diabetes and obesity.^37^, ^38^

Tanycytes appear to be very versatile cells and express a range of receptors for endocrine and paracrine signals (eg, GPR50, TSH‐R, FGFR1 and NMU‐R). More specifically, tanycytes are important in the regulation of body fat mass and leptin responsiveness.^21^, ^22^, ^23^, ^39^ Tanycytes could be the initial target cells for anti‐obesity effect exerted in the brain by leptin, a peptide released from fat tissue in relation to fat mass that may reach tanycytes via the blood, as suggested previously.^23^ IL‐6 produced in the brain may exert a similar anti‐obesity effect after reaching its receptor on tanycytes via the CSF. The leptin receptor shares similarities with class 1‐cytokine receptors, such as IL‐6Rα, utilising the same Janus kinase‐STAT signalling pathway, and the ligands leptin and IL‐6 are also related chemically.^31^ It remains to be investigated whether CSF IL‐6 and blood leptin can potentiate each other's effects on tanycytes.

IL‐6 injected systemically, which could be assumed to only reach tanycytes via the blood circulation from the median eminence, did not induce an increase in pSTAT3‐immunoreactivity in these cells. As expected and in agreement with earlier studies,^23^ leptin induced pSTAT3 in the ARC both when given systemically and i.c.v. STAT3 is a well‐known mediator of the biological effects of IL‐6.^31^, ^40^ Therefore, it is possible that ventricular but not systemic IL‐6 plays a role in regulating biological functions of tanycytes. Further investigations would be required to determine the precise location of IL‐6Rα on tanycytes.

In relation to our present findings of functional IL‐6Rα, metabolically active tanycytes, it is of interest that i.c.v. administration of IL‐6 was found to decrease body fat mass, increase energy expenditure and thermogenesis in mice.^6^, ^10^, ^12^, ^19^, ^41^ No such effect was observed after systemic administration of IL‐6 at a similar^6^ or much higher (Wallenius V, Wallenius K, Feldt J, Wernstedt I, Jansson JO, unpublished observation) dose.

In the present study, we used two different markers for tanycytes: DARPP‐32 and vimentin. At the bottom of 3V, we found extensive and similar immunoreactivity with both markers, a finding that is in agreement with the well‐known presence of tanycytes in this area.^22^ Vimentin is an intermediate filament protein that is expressed in mesenchymal cells. In addition, vimentin binds to smooth muscle cells lining capillaries. DARPP‐32, on the other hand, binds to a dopamine intracellular second messenger protein, which is found in tanycytes. The DARPP‐32 antiserum appears to be more specific for tanycytes, whereas vimentin stains a wider range of cells.^25^, ^26^, ^42^ In the present study, the immunoreactivity pattern of the two antibodies was very similar at the bottom of the 3V. This led us to conclude that both antibodies stain tanycytes in this area and we mainly used vimentin as a tanycyte marker in our study.

In the present study, we found that some cells below the ventricular lining cells at the bottom of the third ventricle were positive for the astrocyte marker GFAP and also positive for IL‐6Rα (see Supporting information, Figure S2). This is in contrast to previous data from other parts of the hypothalamus (eg, the PVN,^14^ LHA^15^ and ARC^16^), as well as the NTS of the hindbrain^43^ where no such co‐localisation between GFAP and IL‐6Rα was found. However, astrocytes are a diverse group of cells and the astrocytes in the subventricular area of the 3V have been reported to differ from other astrocytes. Instead, the subventricular astrocytes may have similarities to tanycytes, such as being positive for DARPP‐32^25^ and IL‐6Rα (present study).

As discussed above, the results of the present study indicate that there are functional IL‐6Rα in cell membranes of mouse tanycytes. Timper et al.^44^ recently reported that i.c.v. administration of IL‐6 can exert biological effects in the absence of IL‐6Rα located in neurones of the hypothalamus. These effects were proposed to be exerted via so called trans‐signalling, a phenomenon that has also been studied extensively elsewhere.^45^, ^46^, ^47^ In the present study, i.c.v. injection of IL‐6 caused a short‐term increase in pSTAT3 in tanycytes, as observed at 5 but not 15 minutes. Timper et al.^44^ found that i.c.v. IL‐6 treatment suppressed food intake, whereas our group has reported increased energy expenditure and decreased body fat mass after i.c.v. IL‐6 injection.^6^, ^10^ Moreover, there are several other differences between our experimental protocol and that of Timper et al.,^44^ including IL‐6 dose, target cell type, diet and type of vehicle. For example, the dose of IL‐6 given i.p. by Timper et al.^44^ was considerably higher (400 ng per mouse) than the dose given by us (80 ng per mouse). This may explain why we did not observe an effect of i.p. IL‐6 treatment. However, the dose of IL‐6 given i.p. in the present study was sufficiently high to induce pSTAT3 staining in some parts of the brain (see Supporting information, Figure S3)

It appears likely that a substantial part of all IL‐6 effects are exerted via integral IL‐6Rα, at least in mice fed normal chow. This would be in agreement with the current paradigm for how hormones in general are acting on cells via cell membrane receptors.^48^ However, further studies are needed to investigate to what extent the effects on tanycytes by IL‐6 are exerted via membrane bound receptors or via trans‐signalling.

There is confirmation that not only the IL‐6Rα, but also the IL‐6 ligand is produced locally in the CNS. We were able to measure substantial IL‐6 mRNA levels in the hypothalamus of animals without inflammation as shown previously.^32^ The levels of IL‐6 mRNA in the CNS appear to be regulated (eg, by GLP‐1 and amylin),^32^, ^35^ further supporting the idea of IL‐6 production being of physiological relevance. The fact that IL‐6 levels are higher in CSF than in serum in many humans further argues for the local production of IL‐6 in the CNS.^20^ So far, it has been difficult to determine exactly in which cells and parts of the hypothalamus that IL6 is produced during health, partly as a result of lack of verified specific IL‐6 antibodies that can used for immunohistochemistry. The results of studies using other techniques suggest that IL‐6 can be produced by microglia or astrocytes in vitro, although this may not reflect the situation in vivo.^35^, ^49^ In summary, less in known about the localisation of the IL‐6 ligand compared to IL‐6Rα in healthy animals.

In summary, the results of the present study show that the ligand binding part of the IL‐6 receptor, IL‐6Rα, is present on β‐tanycytes at the bottom of the 3V. These cells have been reported as being of importance in the regulation of energy balance and body fat mass and, recently, they were hypothesised to be a frontline target cell for the metabolic effects of leptin.^39^ Tanycytes show pSTAT3‐immunoreactivity, similar to leptin, after i.c.v., but not i.p., administration of IL‐6, indicating that IL‐6 produced locally in the brain could be of importance for these cells. This lends new support to our hypothesis that locally produced IL‐6 in the brain plays an important role in the regulation of metabolism.
