# Supplementary material for: Functional interleukin‐6 receptor‐α is located in tanycytes at the base of the third ventricle
Source: J Neuroendocrinol. 2017 Dec 12;29(12):e12546. doi: 10.1111/jne.12546 (PMC5852644; doi:10.1111/jne.12546)

Figure S1 - IL-6Rα immunoreactivity (SC-660) is located to vimentin-positive cells in the median eminence and arcuate nucleus.

Immunohistochemistry showing vimentin-immunoreactivity in green, interleukin-6 receptor α-(IL-6Rα) immunoreactivity in red, and DAPI (nuclear staining) in blue shown in coronal cross-sections of parts of the third ventricle (3V) of the mouse brain. Using a second antibody against IL-6Rα (SC-660), we were able to replicate the results found in the main paper (Figs 1-2). Overview of the area (A) and magnifications of parts of the 3V facing the Arcuate Nucleus (ARC) (B), Median Eminence (ME) (C), 3V lateral wall (D) and top of 3V (E) showed IL-6Rα-immunoreactivity mainly located to vimentin-positive cells at the base of 3V. Orange arrowheads (B-C) indicate examples of cells displaying both vimentin- and IL-6Rα-immunoreactivity. Vimentin-immunoreactive cells in the lateral wall (D) and top (E) of the 3V showed little to no IL-6Rα-immunoreactivity (white arrowheads). Images were obtained using the confocal microscope system described in Material and Methods. Scale bars for overview = 100 µm, close-up = 10 µm.

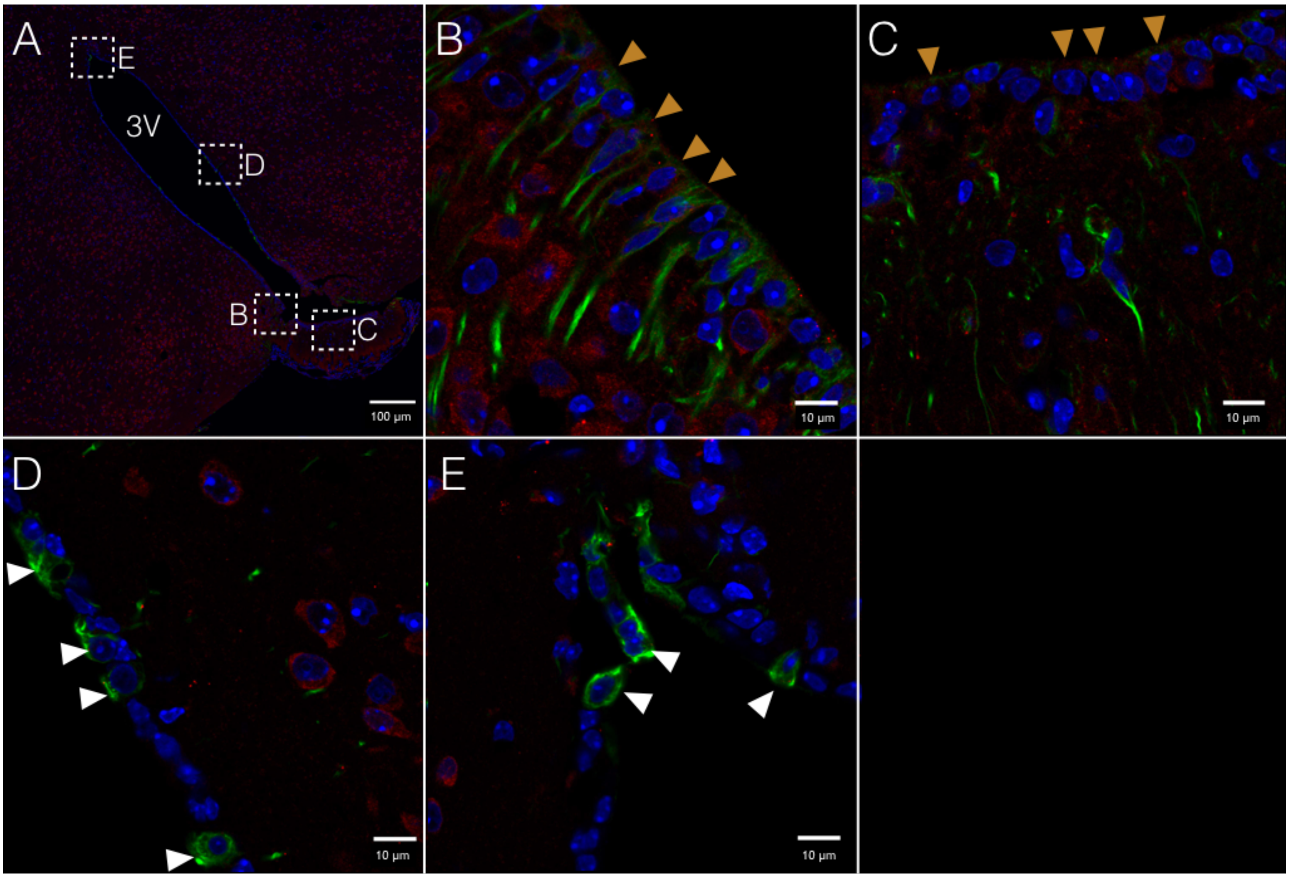

Figure S2 - IL-6Rα immunoreactivity is located to GFAP-positive subependymal cells in the median eminence and arcuate nucleus.

Immunohistochemistry showing interleukin-6 receptor α- (IL-6Rα) immunoreactivity in green, GFAP-immunoreactivity in red, and DAPI (nuclear staining) in blue shown in coronal cross-sections of parts of the third ventricle (3V) of the mouse brain. Overview of the area (A) and magnifications of parts of the 3V facing the Arcuate Nucleus (ARC) (B), Median Eminence (ME) (C) showed IL-6Rα-immunoreactivity on GFAP-immunoreactive cells. White arrows (B-C) indicate examples of such cells displaying both GFAP- and IL-6Rα-immunoreactivity. Of the GFAP-immunoreactive cells, ~45% were also IL-6Rα-immunoreactive (D). Images and cell counting of two animals and two slices per animal were obtained using the confocal microscope system described in Material and Methods. Scale bars for overview = 100 µm, close-up = 25 µm.

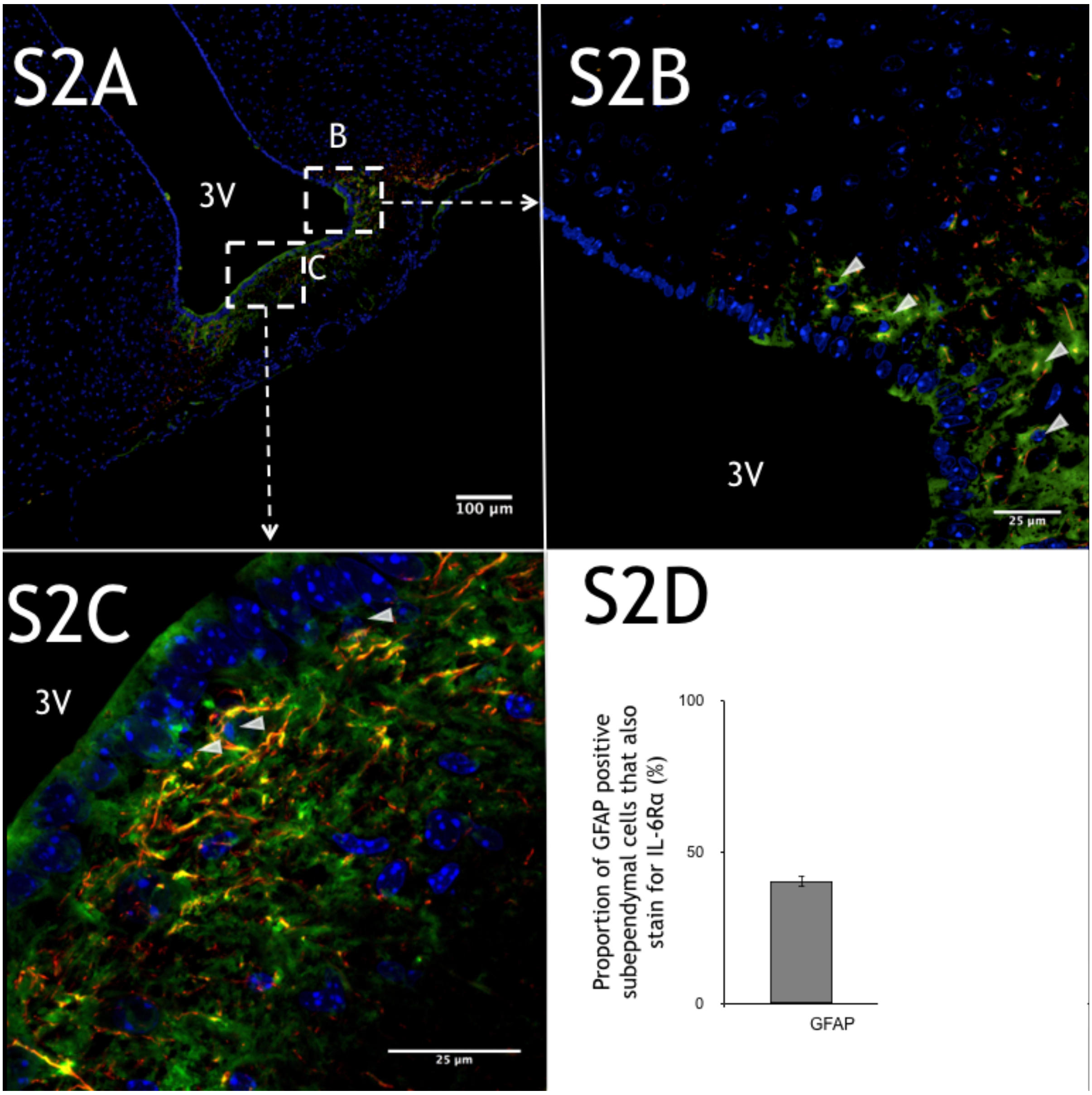

Figure S3 - Peripheral IL-6 administration induces pSTAT3-immunoreactivity in the subfornical organ (SFO) after 15 min.

Immunohistochemistry showing vimentin-immunoreactivity in green, pSTAT3-immunoreactivity in red, and DAPI (nuclear staining) in blue in coronal cross-sections of dorsal parts of the third ventricle (d3V) of the mouse brain. Overview of the area (A) and magnification (B) showed pSTAT3-immunoreactivity in the subfornical organ (SFO) 15 min after i.p. administration of IL-6. White arrowheads show examples of cells in SFO with pSTAT3-immunoreactivity. Images were obtained using the confocal microscope system described in materials and methods. Scale bars for overview = 100 µm, close-up = 10 µm.

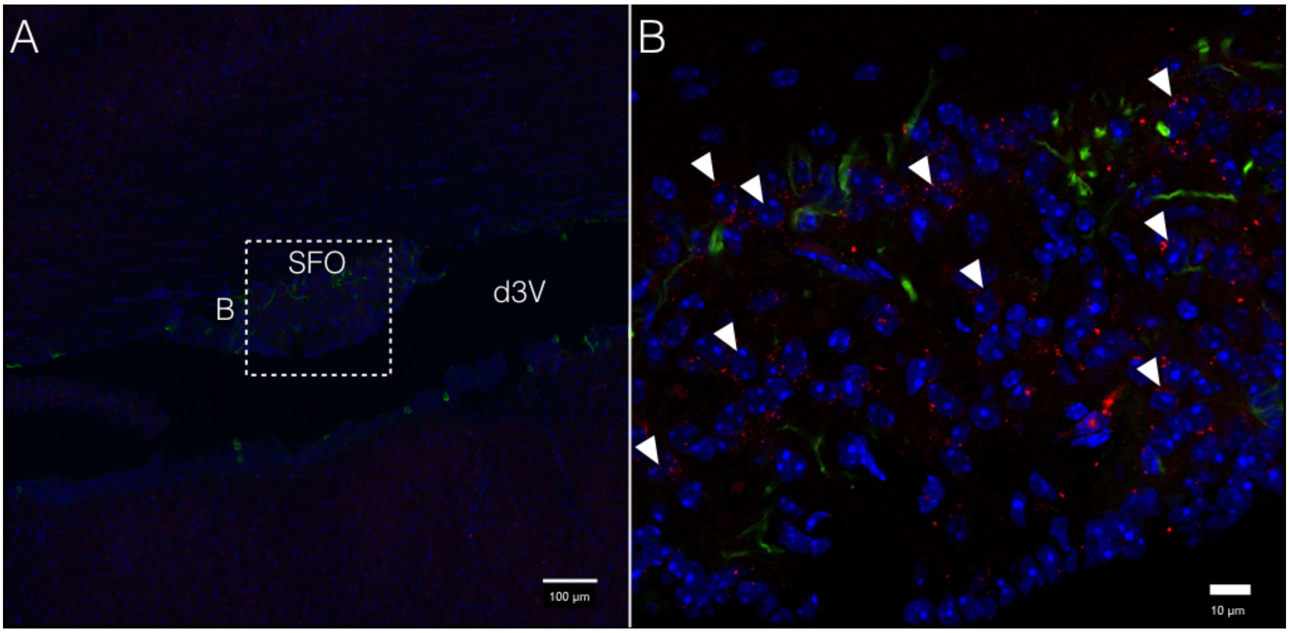

Supplement: Supplementary file 1 [file JNE-29-na-s001.pdf]
